# The Effect of Intercropping with Different Leguminous Green Manures on the Soil Environment and Tea Quality in Tea Plantations

**DOI:** 10.3390/microorganisms12081721

**Published:** 2024-08-21

**Authors:** Pinqian Zhou, Mengjuan Chen, Qiang Bao, Hua Wang, Yuanjiang Wang, Haiping Fu

**Affiliations:** Tea Research Institute, Hunan Academy of Agricultural Sciences, Changsha 410125, China; hncyszpq@hunaas.cn (P.Z.); hnndcmj@163.com (M.C.); hncysbq0703@hunaas.cn (Q.B.); wh13874935124@163.com (H.W.)

**Keywords:** tea, leguminous green manure intercropping, tea quality, rhizosphere bacterial community, soil enzyme activity

## Abstract

Intercropping with green manure is a soil-sustainable cultivation practice that has demonstrated positive impacts on tea growth and the soil environment in tea plantations. Nevertheless, research examining the effect of leguminous green manure varieties in tea plantations is scarce. This study aimed to analyze the tea quality and soil environment components in response to intercropping with three distinct leguminous green manures, *Cassia sophera* cv. Chafei 1 (CF), *Sesbania cannabina* (Retz.) Pers. (SC), and *Chamaecrista rotundifolia* (Pers.) Greene (CR), with 70% chemical fertilizer, and compare them to non-intercropped green manures with 100% chemical fertilizer (CK) in tea plantations. The findings indicated that intercropping with SC increased the amino acids content of tea leaves, the soil organic carbon (SOC), the soil acid phosphatase (ACP), the soil acid protease (ACPT), and the bacterial diversity compared to the CK treatment. Intercropping with CR improved the ACP activity and bacterial diversity while intercropping with CF improved the polyphenols. Proteobacteria, Acidobacteria, Actinomycetes, and Firmicutes were identified as the dominant bacterial taxa in tea plantations with intercropped green manure. A strong positive correlation was indicated between the SOC contents and the amino acids content in tea leaves after intercropping. A canonical correspondence analysis indicated significant associations between the ACP and the urease activity, and between the ACP and ACPT, and both were closely linked to SC. This finding provides an explanation that intercropping with SC may positively affect tea quality by influencing the SOC content, the soil enzyme activity, and the soil bacterial diversity. Green manure intercropping may replace part of chemical fertilizers, improve the soil environment in tea gardens, and enhance the quality of tea. These findings offer a theoretical reference for selecting leguminous green manure and advancing the sustainable development of tea plantations.

## 1. Introduction

Tea is the most popular beverage and is among the top three non-alcoholic drinks worldwide [1]. Secondary metabolites in tea plants, for example, polyphenols, theanine, and caffeine, are closely associated with tea quality [2]. Similarly, tea quality is closely related to the tea variety, the ecological environment, the cultivation model, processing, and extraction [3]. Adopting an efficient cultivation model can extend the production life of tea plantations [4].

Green manure intercropping is a soil-sustainable cultivation model that has progressively been applied to various crops in recent years [5,6,7,8]. Green manure intercropping and its incorporation into fields can stimulate soil microbial activity by providing nutrients to plants. The organic matter supplied from green manures and plant residues induces short-term and medium-term responses in microbial populations by affecting the soil temperature, pH, moisture, and fertility [9,10]. Green manure is often divided into non-leguminous green manure and leguminous green manure. Non-leguminous green manures include ryegrass, buckwheat, radishes, etc. Most non-leguminous green manures are upright, with well-developed root systems and strong enrichment and conversion capabilities [11,12]. Leguminous green manures include sesbania, astragalus, soybeans, and so on. Leguminous green manures are rich in nitrogen, and their roots contain rhizobia. They can fix nitrogen in the atmosphere together with the rhizobia [13,14]. After applying leguminous green manures, the soil nitrogen is generally enriched. Leguminous green manures are associated with an increased soil nitrogen content, and therefore, they change the microbial community structure and activity [10,15]. Tea plants are important economic leaf crops with a high demand for nitrogen [16]. Green manure intercropping in tea gardens can effectively improve the soil status of tea plantations, as well as enhance the soil bacterial flora and the quality of the tea [14]. Leguminous plants, such as soybeans, *Astragalus sinicus* L., *Sesbania cannabina* (Retz.) Pers., and *Chamaecrista rotundifolia* (Pers.) Greene and ‘Chafei 1’, are typically used in various crop fields or tea plantations [6,17,18,19]. Notably, intercropping with leguminous plants improves soil fertility. Indeed, intercropping with soybeans and *Astragalus sinicus* L. can increase the content of ammonium nitrogen, whilst *Astragalus sinicus* L. intercropping specifically increases the ammonium nitrate levels [20,21]. It has been well documented that intercropping with leguminous plants improves tea quality. For instance, intercropping with soybeans and *Astragalus sinicus* L. can improve the content of theanine and soluble sugar [10,17], while pea intercropping promotes amino acid metabolism and flavonoid biosynthesis, thereby optimizing tea quality [22]. Intercropping changes the composition of bacterial flora in tea plantations, while intercropping with soybeans significantly increases the relative abundance of beneficial bacteria such as Rhizobia, Saccharomonas, and Mycobacteria. *Pseudogulbenkiania*, *SBR1031*, and *Burkholderiaceae* had correlations with the soil physicochemical properties and tea quality traits [23]. Intercropping with the soybean ‘Hefeng47’ in the summer and wild peas in the winter significantly affected the bacterial community in tea plantations, affecting the abundance of Bacillus [17]. The bacterial community in tea plantations shifts from oligotrophy to eutrophy after intercropping with leguminous green manure soybeans (*Glycine max* L.) [24]. Overall, the tea intercropping system plays an instrumental role in improving the soil environment in tea plantations and tea quality components in tea leaves.

A study reported that 30% of tea gardens in China are over-fertilized with chemical fertilizers [25], eventually leading to soil acidification and consolidation, heavy metal accumulation, and a low utilization rate of nitrogen fertilizers, thereby posing health risks [26]. While leguminous green manure intercropping can effectively enhance the physical properties and chemical properties of the soils and the tea quality, the effects of different leguminous green manure treatments vary, and their specific effects warrant further investigation. This study determined the chemical composition of tea leaves, measured the organic carbon content and enzyme activity in tea garden soil, and employed 16S to analyze changes in the bacterial flora after leguminous green manure intercropping in tea plantations. From intercropping with three leguminous green manures, the one exerting the optimal effect on the tea quality and soil environment was selected for future promotion.

## 2. Materials and Methods

### 2.1. Site Selection, Soil Sampling, and Basic Soil Conditions

The experimental tea farm is located at Hunan Tea Research Institute, Gaoqiao Town, Changsha County, Hunan Province, China (E113°20′23.8″, N28°28′40.9″), at an altitude of 52.6 m. The tea variety under study, ‘Huangjincha 2’, was planted in 2017 with a double-row, double-plant configuration and a row spacing of 160 cm. Green manure intercropping was initiated in 2018 and has been conducted annually since then. The soil samples were collected by shaking off the loose soil attached to the tea roots. The soil tightly attached to roots was classified as rhizosphere soil. The roots and residues visible to the naked eye in the rhizosphere soil were removed, and the soil was divided into two parts. One portion was stored at −80 °C for soil bacterial diversity and the other portion was used for soil enzyme activity determination. The soil type was yellow soil, with the following fertility characteristics at a depth of 0~20 cm: pH 4.84. The tea leaf samples, one bud and two leaves, were picked in spring, freeze-dried, and used for biochemical quality analysis.

### 2.2. Experimental Treatments

The experiment consisted of four treatments: (1) intercropping with no leguminous green manure and applying 100% chemical fertilizer (450 kg/ha urea, CK); (2) intercropping with leguminous tea *manure Cassia sophera* cv. ‘chafei 1’ (CF) and applying 70% chemical fertilizer; (3) intercropping with *Sesbania cannabina* (Retz.) Pers. (SC) and applying 70% chemical fertilizer; and (4) intercropping with *Chamaecrista rotundifolia* (Pers.) Greene (CR) and applying 70% chemical fertilizer. Each treatment was replicated 3 times across 12 plots, with each experimental plot covering an area of 50 m^2^. Intercropping with leguminous green manure was carried out by cutting and returning to the field immediately after ditching the soil. All other field management measures were kept consistent for each treatment.

### 2.3. Determination of Tea Biochemical Quality

The contents of amino acids, polyphenols, and caffeine were analyzed using ultraviolet spectrophotometry according to national standard [27,28,29], and water extracts content was detected by boiling water extraction according to national standard [30].

### 2.4. Determination of Soil Chemical Properties

The content of soil organic carbon (SOC) was determined using an elemental analyzer (Vario ELIV). There was no difference in the total nitrogen, total phosphorus, total potassium, alkaline nitrogen, available phosphorus, or available phosphorus contents among the four treatments, which were 1.27 g/kg, 0.45 g/kg, 14.9 g/kg, 89.0 mg/kg, 4.07 mg/kg and 143.0 mg/kg, respectively.

### 2.5. Soil Enzyme Activity Determination

The activities of soil catalase (CAT), soil sucrase (SC), soil acid phosphatase (ACP), soil acid protease (ACPT), and soil urease were determined using soil microplate fluorescence spectrophotometry.

### 2.6. DNA Sequencing Analysis of Soil Bacteria

The bacteria communities in soil samples treated with organic and inorganic fertilizers were analyzed using Illumina Hiseq sequencing. Bacterial DNA was extracted using the OMEGA soil DNA kit, and the quality of the extracted DNA was checked. The 515F/806R primers were used to amplify the V4 region of the 16S rRNA [31] to identify bacteria diversity in soil. Polymerase chain reaction (PCR) was used to amplify DNA fragments, with cycling conditions based on previous studies [6]. Purified PCR products were used to construct DNA libraries. After confirming DNA quality, sequencing was performed using the Illumina HiSeq platform.

### 2.7. Bioinformatics and Statistical Analysis

The bioinformatics and statistical analysis methods are detailed in our prior research [6]. Data variance was analyzed using SPSS software (version 22.0), utilizing Duncan’s multiple range test with a significance level of *p* < 0.05. Means and standard deviations (SDs) were computed from three independent biological replicates. Data processing and visualization were conducted using GraphPad Prism.

## 3. Results

### 3.1. Effects of Intercropping with Leguminous Green Manure on Tea Quality Indicators, Soil Organic Carbon, and Enzyme Activity in Tea Plantations

**Figure 1:**
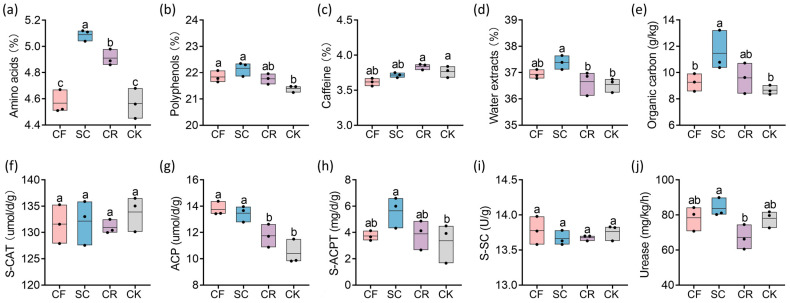
Effects of intercropping leguminous green manure on tea quality indicators, soil organic carbon, and soil enzyme activities. (**a**–**d**) The content of amino acids, polyphenols, caffeine, and water extracts in tea leaves. (**e**) Organic carbon. (**f**–**j**) The activities of CAT, ACP, ACPT, SC, and urease. Different letters indicate significant differences among the samples (*p* < 0.05).

Intercropping with different leguminous green manures influences tea quality indicators, including amino acids, polyphenols, caffeine, and water extracts. The most significant change was observed in amino acids. The amino acids content in tea leaves from SC and CR intercropped was significantly higher than that in CK. Specifically, the amino acids content in SC was 11.6% higher compared to CK. Similarly, the polyphenols content in SC-intercropped tea plantations was significantly higher than that in CK. However, no significant differences were observed in caffeine content among the different green manure intercropped tea plantations. Lastly, water extracts content was significantly higher in SC-intercropped tea plantations than in CK (Figure 1a–d).

Leguminous green manure intercropping affects SOC content in tea gardens. The SOC content in SC-intercropped plots was significantly higher than that in CK (Figure 1e). Specifically, the SOC content in SC was 32.23% higher compared to CK. No significant differences were noted between CF and CR intercropped plots compared to CK. Intercropping with leguminous green manure significantly impacted soil acid enzyme activity. Intercropping with CF, SC, and other tall green manures increased the ACP activity. Specifically, SC intercropping increased S-ACPT activity, whereas CR intercropping reduced soil urease activity compared to SC. However, intercropping with these three green manures had a marginal effect on catalase and soil sucrase activities (Figure 1f–j).

### 3.2. Effect of Leguminous Green Manure on Taxa and Microbial Community Structure

**Figure 2:**
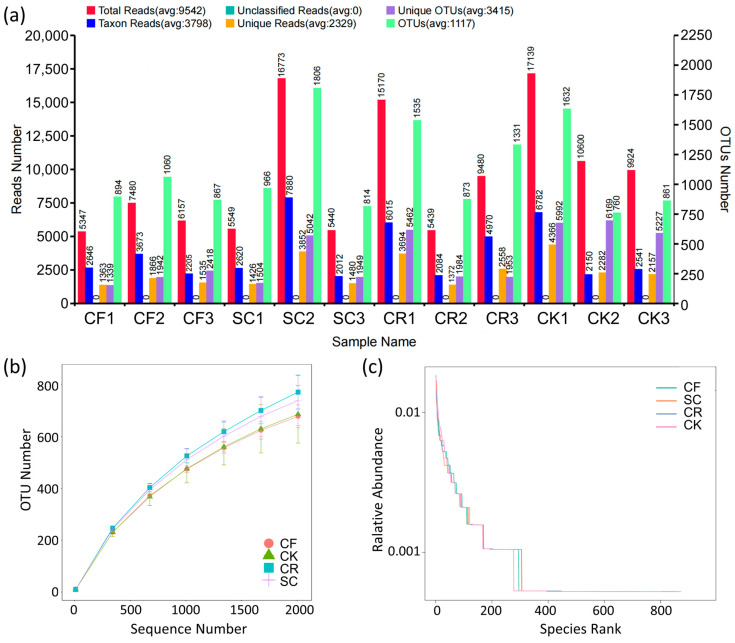
Analysis of relative abundance, Alpha index dilution, and diversity curves of bacteria across different treatments: (**a**) Relative abundance of phylum across different treatments. (**b**) Dilution plot of the Alpha index in soil across treatments. (**c**) Rank abundance in soil across treatments.

Microbial taxonomic responses to different leguminous green manure applications are illustrated in Figure 2a. Operational taxonomic units (OTUs) were 940, 1195, 1246, and 1084, with corresponding total reads of 6328, 9254, 10,030, and 12,554, taxon reads of 2841, 4170, 4356, and 3824, and unique reads of 1588, 2252, 2541, and 2935 for CF, SC, CR, and CK, respectively. Despite the relatively lower OTUs in the three green manures, the ratio of OTUs to total reads was 14.86%, 12.92%, 12.43%, and 8.64% for CF, SC, CR, and CK, respectively. As anticipated, CK exhibited the highest total number of reads and OTUs, as well as the lowest OUT to total reads ratio (Figure 2a). The Alpha index rarefaction curve displayed that CR had the highest bacterial diversity index, while CK and CF had the lowest bacterial diversity indices (Figure 2b). Bacterial diversity curve analysis depicted that SC and CR had the highest diversity, followed by CF and CK (Figure 2c).

**Figure 3:**
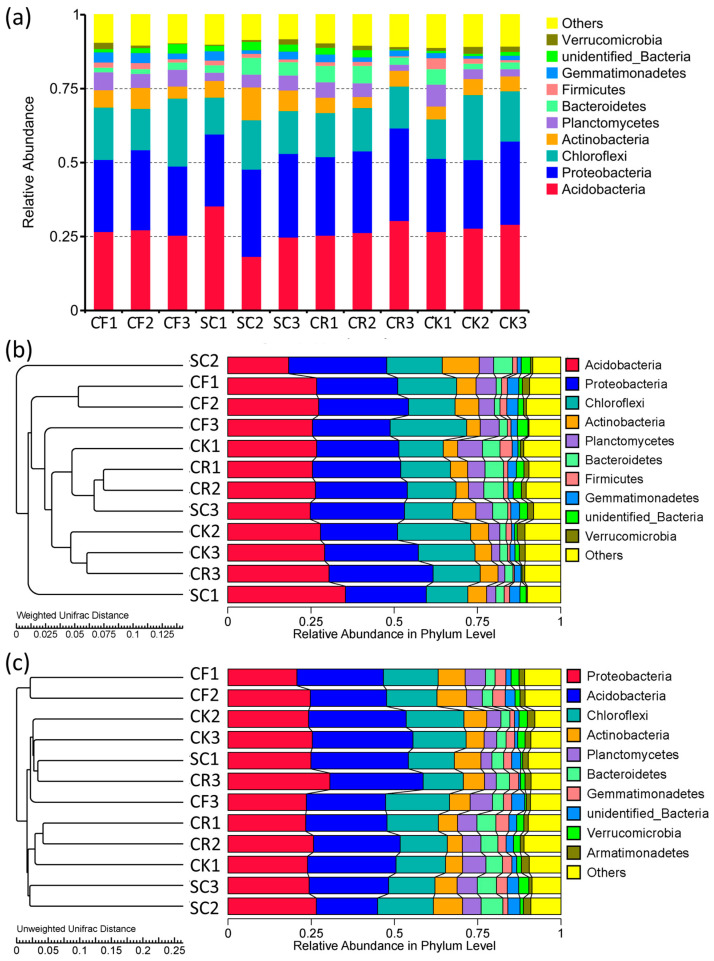
Relative abundance of major bacterial taxa. (**a**) Relative abundance histogram of phylum-level dominant bacteria. Weighted (**b**) and unweighted (**c**) pairwise method of clustering trees based on arithmetic mean weighted unipartite distance.

Analysis of the relative abundance distribution in tea garden soils under different leguminous green manure treatments revealed Acidobacteria, Proteobacteria, Chloroflexi, Actinobacteria, and Planctomycetes as the dominant phyla (Figure 3a). The relative abundance in SC was higher than in CF, CR, and CK. with CK soil showing the lowest relative abundance. Acidobacteria and Chloroflexi relative abundance in CK soil was higher compared to other treatments, whereas Proteobacteria abundance was higher in SC and CR compared to CF and CK. The weighted single fractal distance cluster tree analysis exposed that SC replicates were the most distant in clustering, while CF replicates were the most similar. CK and CF had a higher abundance of Acidobacteria and a lower abundance of Chloroflexi compared to CR (Figure 3b). Unweighted single fractal distance cluster tree analysis showed a close clustering relationship between CK and CF, with CK exhibiting a significantly higher abundance of Acidobacteria compared to other treatments. CF had the highest relative abundance of Chloroflexi, whilst SC and CF had a high relative abundance of Actinobacteria compared to CK and CF. Finally, the relative abundance of Bacteroidetes was higher in CR and SC compared to CK (Figure 3c).

### 3.3. Composition of Bacterial Community

**Figure 4:**
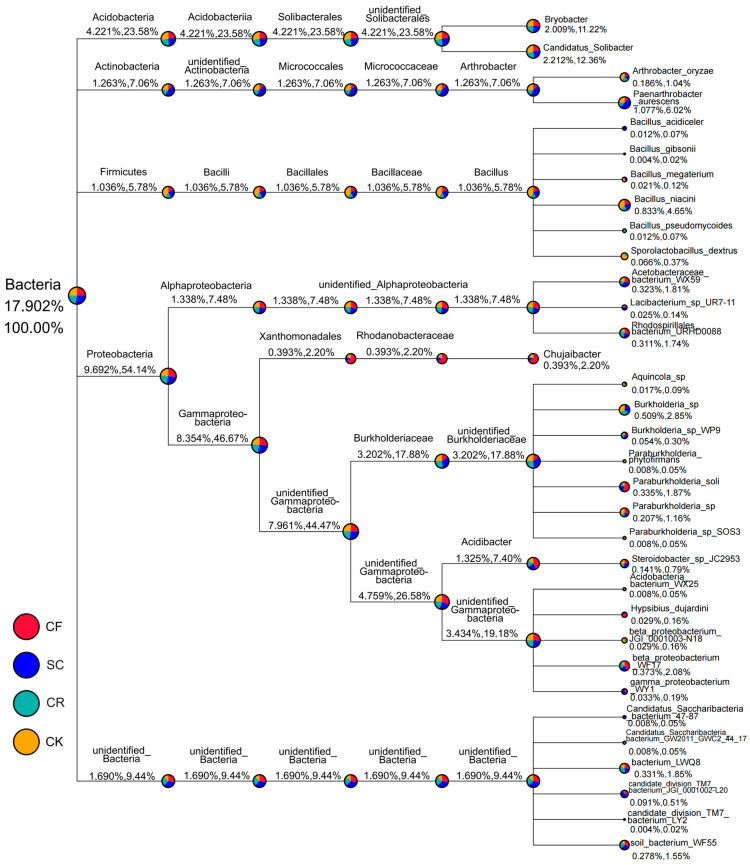
Relative abundance presented as a percentage and a taxonomic tree. Each color-coded sector represents a specific treatment and microbial composition; numbers below the taxonomic name denote the relative abundance at that classification grade.

The distribution of bacterial communities under different leguminous green manure patterns (Figure 4) illustrated differences in the composition of abundant bacteria. The taxonomic tree of the top 10 genera by relative abundance indicated that Proteobacteria comprised 54.14% of the total, with 46.67% attributed to Gammaproteobacteriaand 7.48% to Alphaproteo. Burkholderiaceae constituted 17.88%. Acidobacteria represented 23.58%, with 11.22% from Bryobacter and 12.36% from Candidatus_Solibacter. Actinobacteria and Firmicutes accounted for 7.06% and 5.78%, respectively, with Paenarthrobacter aurescens (6.02%) and Bacillus_niacini (4.65%) being the most abundant species. Notably, the abundance of Actinobacteria was substantially higher in SC compared to other treatments (Figure 4).

### 3.4. Phylogenetic Tree

**Figure 5:**
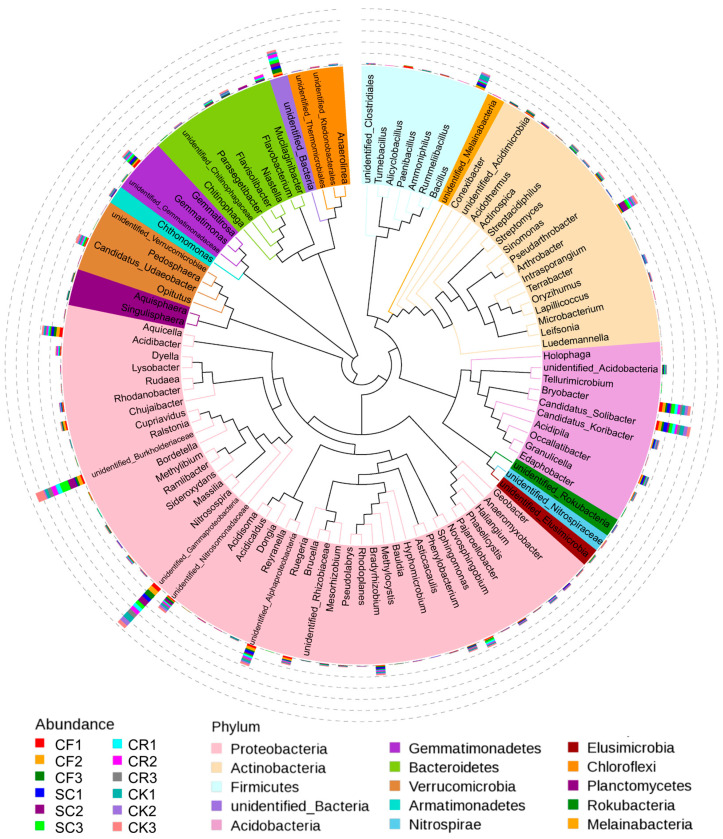
Phylogenetic tree presenting horizontal species: branch color and fan shape represent the corresponding phyla and abundance, respectively, with genes displayed at different taxonomic levels; the stacked bar graph outside the sector ring denotes the abundance distribution of the genus.

Phylogenetic relationships at the genus level showed that Proteobacteria was the most abundant phylum, followed by Actinobacteria. Burkholderiaceae, Bryobacter, and Candidatus_Solibacter were abundant across all treatments and soil layers. Proteobacteria primarily comprised of *Acidibacter*, *Brucella*, *Bradyrhizobium*, and *Sphingomonas*, whereas Actinobacteria was mainly composed of *Arthrobacter*, *Streptacidiphilus*, and *Sinomonas*. Bacillus was the predominant genus within *Firmicutes*. SC had a high abundance of *Arthrobacter*, while CF showed a high abundance of *Acidibacter*. The phylum Gemmatimoadetes included *Gemmatimonas* and *Gemmatimorosos*, with *Gemmatimonas* being the predominant genus (Figure 5).

**Figure 6:**
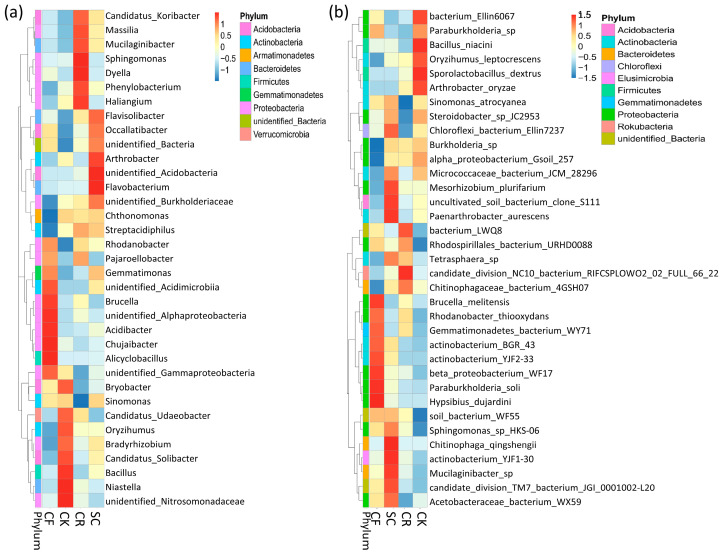
Heatmap portraying clustering of genus and species abundance; clustering based on genus (**a**) and species (**b**) classification. Horizontal axes represent classification positions, whereas vertical clustering is based on the top 35 genera samples.

Bacterial clustering was analyzed at both the genus and species levels, as demonstrated in the heat map. The heatmap at the genus level revealed the most abundant bacteria in CF were *Brucella*, *Acidibacter*, *Chujaibacter*, and *Alicyclobacillus*, with the first three bacteria belonging to the Acidobacteria phylum. CK was abundant in *Bacillus* and *Niastella*, while CR was dominated by *Sphingomonas*, *Dyella Phenylobacterium*, and *Haliangium*, all within the Acidobacteria phylum. In SC, *Arthrobacter* and *Flavobacterium* were the dominant bacteria genera (Figure 6a). Moreover, CF’s most abundant bacteria included *beta_proteobacterium_WF17*, *Paraburkholderia_soli*, and *Hypsibius_dujardini*, all belonging to the Proteobacteria phylum. Conversely, the most abundant bacteria in SC were *Chitinophaga_qingshengii* and *Paenarthrobacter_aurescens*. Compared to other treatments, CR was not highly enriched in bacterial species (Figure 6b).

### 3.5. Functional Prediction of Soil Bacterial Communities

**Figure 7:**
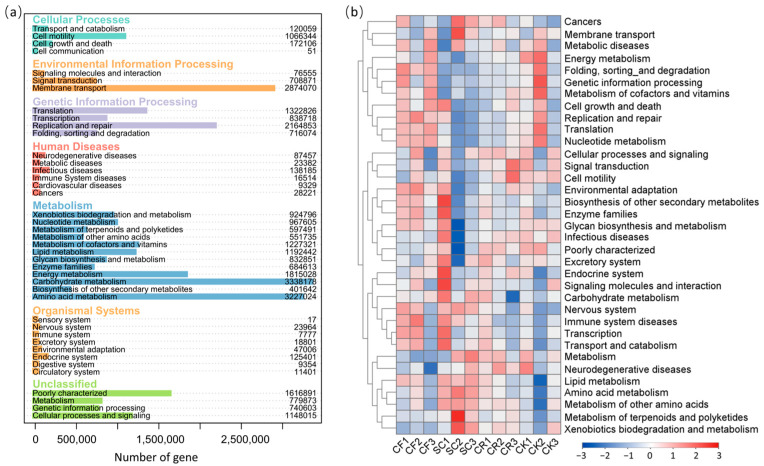
Prediction of the differential function of bacterial communities. (**a**) Gene functions annotation on KEGG. (**b**) Heatmap of gene functional predictions of samples based on KEGG level 2.

The PICRUSt2 algorithm was used to retrieve data from the KEGG annotation database for predicting bacterial functions. A total of 41 KEGG categories were expected. Carbohydrate metabolism, amino acid metabolism, and membrane transport were the three most enriched KEGG pathways (Figure 7a). The pathway enrichment across different treatments was further analyzed. Notably, carbohydrate metabolism, amino acid metabolism, lipid metabolism and metabolism of other amino acids were enriched in SC but not in CK. Environmental adaptation was enriched in CF but not in CK (Figure 7b).

### 3.6. Correlation Analysis of Intercropping with Leguminous Green Manure in Tea Garden on Tea Quality Indicators, Soil Properties, and Soil Bacterial

**Figure 8:**
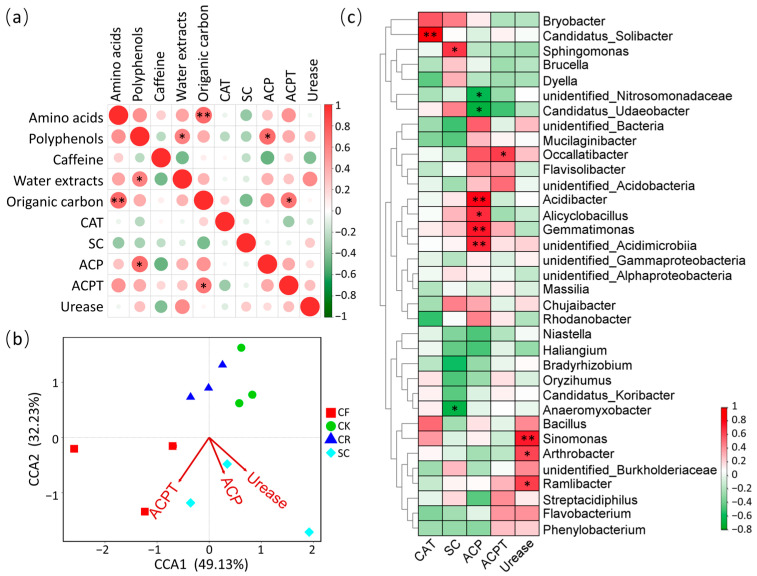
Correlations between tea quality indicators and soil properties. (**a**) Correlations between tea quality indicators, soil organic carbon, and enzyme activity. (**b**) Correlations between enzyme activity and different treatment by CCA. (**c**) Correlations between enzyme activity and bacteria at the genus level. Pearson’s correlation, * *p* < 0.05, ** *p* < 0.01.

After leguminous green manure intercropping, amino acids content in tea leaves was highly significantly positively correlated with SOC level (*p* < 0.01), and S-ACPT was significantly positively correlated with SOC content (*p* < 0.05). Moreover, the content of water extracts showed a significant positive correlation with polyphenols content and ACP (*p* < 0.05), whereas correlations with other indicators were not significant (Figure 8a). Furthermore, CCA analysis was performed to explore relationships among different treatments, bacterial communities, and enzyme activities at the genetic level. The results exposed that ACP was highly correlated with urase and ACPT, implying a positive association with SC (Figure 8b). Spearman correlation analysis identified a highly significant positive correlation between CAT and *Candidatus_Solibacter*, a significant positive correlation between SC and *Sphingomonas*, and a significant negative correlation between SC and *Anaeromyxobacter*. Additionally, ACP showed a highly significant positive correlation with *Acidibacter* and *Gemmatimonas*, a significantly positive correlation with *Alicyclobacillus*, and a significantly negative correlation with *Candidatus_Udaeobacter*. Finally, ACPT showed a significantly positive correlation with *Occallatibacter*, while urease had a highly significantly positive correlation with *Sinomonas* and a significantly positive correlation with *Arthrobacter* and *Ramlibacter* (Figure 8c).

## 4. Discussion

### 4.1. Effects on the Formation of Tea Key Quality Components in Tea Leaves

Leguminous intercropping represents an environmentally friendly strategy for advancing sustainable modern agriculture [32,33,34]. Long-term excessive application of nitrogen fertilizers has led to environmental pollution, aggravated soil acidification, reduced microbial diversity, and diminished plant efficiency of utilization fertilizers [35,36]. Approximately 30% of tea plantations in China are over-fertilized with chemical fertilizers. Reducing the use of chemical fertilizers can decrease total nitrogen and total phosphorus inputs in the short term and lower tea garden management costs. Replacing chemical fertilizers with organic fertilizers is the best long-term strategy for reducing fertilizer application [25]. Our research showed a positive effect on tea quality by substituting 30% of chemical fertilizers with leguminous green manure through intercropping in tea plantations. Previous studies have described that leguminous green manure intercropping significantly promotes the synthesis of soluble sugars, amino acids, and EGCG in tea leaves [14]. Our results showed that intercropping with SC and CR significantly increased amino acids content (Figure 1a). The high amino acids content in tea can enhance the freshness profile, thereby improving its quality and price [37,38]. Importantly, intercropping SC and CF significantly increased polyphenols content, and specifically, SC intercropping increased the water extracts content (Figure 1b,d), thereby improving the concentration and flavor profile of tea [3]. In summary, these results demonstrate that leguminous green manure intercropping has the potential to improve tea quality. Although SC, CR, and CF are all leguminous plants, their effects on improving tea’s chemical composition vary, as evidenced by significant differences in tea quality among different intercropped plants used as green manure.

### 4.2. Effect of Intercropping Leguminous Green Manure on Soil Bacterial Community

Soil bacteria are crucial for soil health and plant growth [39]. This study analyzed the changes in bacterial community structure induced by intercropping with green manure. The results revealed that intercropping CR, SC, and CF effectively improved the diversity and abundance of soil bacteria, with SC and CR showing optimal effects (Figure 2). Proteobacteria, Actinobacteria, and Firmicutes are the predominant phyla in tea garden soil, aligning with findings from soybean intercropping in tea plantations [14]. Proteobacteria constituted a numerically significant microbial group in leguminous green manure intercropping and thus serve as valuable indicators of soil nutrient status, typically dominating in nutrient-rich soils and Acidobacteria predominating in nutrient-poor soils [40,41,42]. The relatively high abundance of Proteobacteria herein reflects adequate soil nutrient levels. These phyla are commonly detected in bacterial communities worldwide. In CF-treated soil, the bacterial communities dominated by Acidobacteria including Brucella and Acidobacterium, increased. Similarly, the abundance of *beta_proteobacterium_WF17*, *Paraburkholderia_soli*, and *Hypsibius_dujardini* increased. In SC-treated soil, the abundance of *Arthrobacter* and *Flavobacterium*, along with *Chitinophaga_qingshengii* and *Paenarthrobacter_aurescens*, were increased (Figure 6b). Different subgroups of acidifying bacteria may play contrasting ecological regulatory functions and mediate changes in plant metabolite levels. For example, Acidobacteria are reported to be involved in the degradation of lignocellulose and cellulose and to be dominant in soils rich in organic matter [43,44]. In contrast, Proteobacteria are syntrophic organisms that promote soil ecological stability by supplying nitrogen [45]. Actinomycetes are implicated in the natural nitrogen cycle and can alter soil chemistry to benefit plant growth [46,47,48]. Thus, different species of leguminous green manure exert varying effects on soil bacterial communities, potentially affecting the structure and composition of the microbial flora.

### 4.3. Mutual Influence of Soil Properties, Soil Bacterial Communities, and Tea Quality Indicators

**Figure 9:**
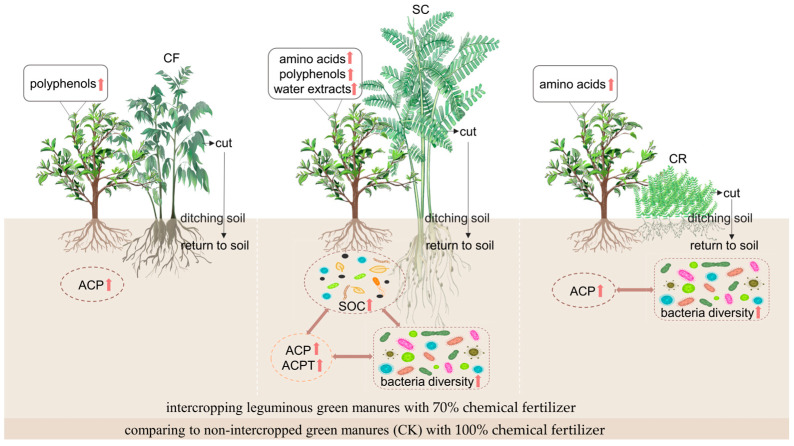
Schematic diagram of tea quality indicators and soil environment after intercropping three different leguminous green manures in the tea plantations. CF: *Cassia sophera* cv. Chafei 1; SC: *Sesbania cannabina* (Retz.) Pers; CR: *Chamaecrista rotundifolia* (Pers.) Greene; SOC: Soil organic carbon; ACP: soil acid phosphatase; ACPT: soil acid protease. The red upward arrow represents a significant increase (*p* < 0.05) compared to CK.

Soil microbial communities are associated with soil enzyme activities and impact plant growth and secondary metabolism [49]. The correlation between soil enzyme activity, soil bacterial population, and tea quality traits in tea plantations using leguminous green manure intercropping has been extensively explored [14,50]. This study described the tea quality indicators, soil enzyme activity and soil bacterial changes after intercropping with three distinct leguminous green manure CF, SC, and CR, comparing them to CK (Figure 9).

SC mulching significantly increases the SOC levels in tea plantations (Figure 1e), contributing to soil improvement. Of note, previous studies have documented that intercropping with leguminous green manure can increase the content of theanine [20], which makes up approximately 50% of amino acids and contributes to the umami flavor of tea [3]. Our results revealed that intercropping SC and CR increased the amino acids content in tea leaves. Indeed, a highly significant positive correlation was identified between the SOC level in the soil and the amino acids content in tea leaves. SOC level has been utilized as an indicator of soil organic matter content [50]. Intercropped with leguminous plants may increase leaf amino acids content by increasing soil organic matter content [51]. Bacterial function prediction revealed that carbohydrate metabolism and amino acid metabolism were enriched in SC treatment (Figure 7), which may have influenced soil carbon content and amino acids level in tea plants. A study on a vegetable field continuously planted for 10 seasons showed that replacing part of the chemical fertilizer with organic fertilizer significantly increased SOC, catalase activity, and urease activity [52]. ACPT was significantly positively correlated with SOC of tea plantations (Figure 8b), in line with previous research results on enzyme activity and biochar applications [53]. Soil enzyme activity is intricately related to microbial activity [54]. Previous studies have shown that different fertilization methods can affect pH, moisture, total N, and SOC content, thereby affecting the structure of soil bacterial communities [19]. Notably, our results showed that ACP activity was significantly higher in CF and SC compared to CK, while ACPT activity was significantly higher in SC than in CK. CCA analysis suggested that ACP was significantly correlated with urease and ACPT activity, with these correlations being particularly pronounced in SC (Figure 8c). Different enzyme activities at the genus level exhibit different correlations with different bacteria. For instance, ACPT showed a significant positive correlation with the abundance of Occallatibacter, a beneficial soil bacterium. ACPT was significantly correlated with SOC content, which was significantly correlated with the amino acids in tea leaves. This finding further suggests that SC may positively affect tea quality by enhancing SOC content and enzyme activity, thereby altering the soil bacterial community. Intercropping with leguminous green manure and returning it to the field offers an effective strategy for replacing chemical fertilizers with organic fertilizers in tea plantations, representing an environmentally friendly cultivation model. We recommend planting green manures like *Sesbania cannabina* (Retz.) Pers. (SC) and *Chamaecrista rotundifolia* (Pers.) Greene to enhance the soil environment of the tea plantations and improve tea quality.

## 5. Conclusions

The effect of intercropping with leguminous green manure in tea plantations was examined, and the results validated its beneficial impact on enhancing soil fertility and tea quality, as well as enriching bacterial communities. Different leguminous green manure varieties exerted varying effects on improving tea quality and soil environment.

## Data Availability

The raw sequencing data files are available in the NCBI SRA database under the project accession no. PRJNA1143986. The data presented in this study are available on request from the author.
